# Selection of superior late‐blooming almond (*Prunus dulcis* [Mill.] D.A.Webb) genotypes using morphological characterizations

**DOI:** 10.1002/fsn3.3370

**Published:** 2023-04-21

**Authors:** Fatemeh Beigi, Ali Khadivi

**Affiliations:** ^1^ Department of Horticultural Sciences, Faculty of Agriculture and Natural Resources Arak University Arak Iran

**Keywords:** almond, breeding, kernel quality, late‐blooming, variation

## Abstract

Almond (*Prunus dulcis* [Mill.] D.A.Webb) is one of the earliest domesticated trees and the evidence dates back to 3000–2000 BC. In the present study, 198 almond seedling origin trees were studied to select late‐flowering genotypes having high kernel quality. Significant variabilities were exhibited among the genotypes investigated based on the recorded traits. Full‐blooming date ranged from mid‐March to mid‐April. The Ward dendrogram clustered the genotypes into two major clusters forming several subclusters. After clustering the genotypes based on the full‐blooming dates, 68 late‐blooming genotypes were recognized and reanalyzed based on the quantitative characters to select the superior ones. Nut‐related characters were as follows: nut length: 22.34–43.05 mm, nut width: 14.07–24.34 mm, nut thickness: 9.21–18.00 mm, nut weight: 1.88–6.62 g, and shell thickness: 2.26–4.59 mm. Kernel‐related characters were as follows: kernel length: 16.73–25.91 mm, kernel width: 8.50–13.64 mm, kernel thickness: 3.56–7.37 mm, and kernel weight: 0.35–1.41 g. Kernel weight was positively and significantly associated with nut weight, kernel thickness, kernel length, kernel width, nut length, and branch leaf width. Thus, these key variables are the main traits accounting for kernel weight, and they should be considered together in breeding with aiming at increasing the kernel weight. Based on ideal values of the important and commercial characters of almond, such as fruit yield, nut weight, shell hardness, kernel shape, kernel weight, and kernel taste, 19 late‐blooming genotypes were promising and are recommended for cultivation in orchards.

## Graphical abstract

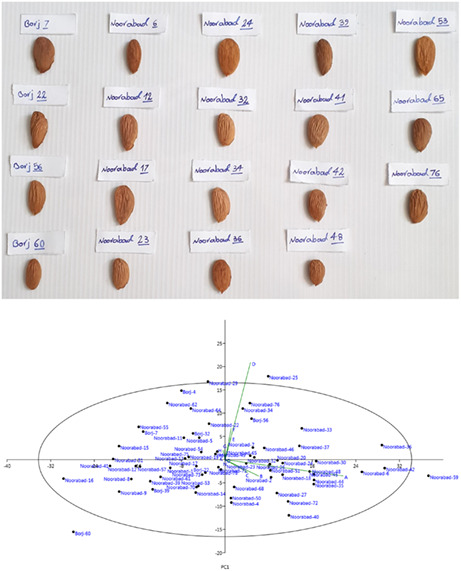

## 1 INTRODUCTION

Almond (*Prunus dulcis* [Mill.] D.A.Webb) is one of the first domesticated fruit trees, dating back to 2000–3000 BC. Mediterranean countries such as Turkey and Syria have been proposed as its origin (Ladizinsky, 1999). The growth and development of almond nut are influenced by hot summer temperature and low winter temperature. The suitable temperature for the development of buds and flowering of almonds is 24°C. Almond growth is not sensitive to the type of soil and thus it can be cultivated in different soils varying from sandy loam to sandy clay, but it prefers deep and fertile soils with proper drainage. The growth and development of this tree are not affected by pH, but it prefers pH 6.50 (Verma et al., 2010).

In various species of *Prunus* genus, such as peach, plum, sweet cherry, sour cherry, and apricot, the edible part includes the flesh (mesocarp), while almond kernel, which is inside the endocarp, is its edible part. So almonds are unique in this respect. Also, the whole part of the almond fruit (seed, mesocarp, and endocarp) can be consumed during maturity, while in most species of the genus *Prunus*, the fruit can only be consumed during maturity. Almond kernels have a high nutritional value and it has a high demand in world markets, and its endocarp (shell), and mesocarp are used as animal feed (Rabinowitz, 2006).

Almonds are usually preferred in the form of shelled nut or consumed as roasted kernels. Also, it can be used as almond milk or oil (Font i Forcada et al., 2011). Almonds are also used in cooking and baking (Sang et al., 2002). Almond kernels are considered as one of the most valuable substances in terms of health and nutrition, and they are free of cholesterol. In total, 100 g of almond kernel contains 575 kcal, 12.20 g of fiber, 26 mg of vitamin E, 949 mg of total fat, 21 g of protein, 670 mg of potassium, 268 mg of magnesium, 484 mg of phosphorus, 265 mg of calcium, and 3.50 mg of iron. Its saturated fatty acid content is low, but it has other important nutrients (Blanca, 2007).

In general, breeding of other plants is easier than fruit trees. The first step for investigating and classifying indigenous and local plant resources is the use of morphological traits (Salim et al., 2018). Success in breeding programs depends on genetic diversity (Arslanoglu et al., 2011). Genetic diversity is used to investigate taxonomic relationships, select promising cultivars and genotypes, and introduce them for use in breeding programs as well as cultivation in orchards (Balkaya & Ergun, 2008).

One of the most important factors limiting the distribution and production of horticultural crops in the world is damage caused by spring frosts. Almond flowers open early, which results in spring frost damages (Imani & Khani, 2011). Therefore, the cultivation and production of this nut in regions with spring frosts are limited (Kodad and Socias i Company, 2004). Therefore, identification and introduction of late‐blooming genotypes and cultivars are one of the most important goals in breeding programs of almonds (Kester & Asay, 1975). Iran is one of the most important almond‐producing countries in the world and it has a long history of almond cultivation as well as rich almond seedling origin trees. In the present study, the seedling‐originated populations were assessed to identify late‐blooming almond genotypes. In the first step, the selected trees were examined in terms of flowering time. In the second step, morphological diversity of the selected genotypes was investigated using the traits related to tree, leaf, and fruit. In the third step, late‐flowering genotypes were identified and then the traits related to their kernel quality were used to select the superior late‐flowering genotypes.

## 2 MATERIALS AND METHODS

### 2.1 Plant material

A total of 198 seedling origin almond trees were selected in the Shazand region of Markazi province, Iran, and were studied for two consecutive years (2021 and 2022), with the aim of selecting superior late‐flowering genotypes in terms of kernel quality. In the first step, the selected trees were examined in terms of full blooming dates (50% of flowers completely open; Sakar, Yamani, Boussakouran, & Rharrabti, 2019; Sakar, Yamani, & Rharrabti, 2019). The control cultivar used was “Tardy‐Nonpareil,” which is a late flowering cultivar. In the second step, morphological diversity of the selected genotypes was investigated using the traits related to tree, leaf, and fruit. In the third step, late‐flowering genotypes were identified and then the traits related to their kernel quality were used to select the superior late‐flowering genotypes. Shazand region is located at latitude 33°57′44″N, longitude 49°25′15″E, and 1913 m height above sea level, with mean annual temperature of 13.90°C and annual precipitation of 320 mm. The genotypes were named based on their studied areas and started with a number. The collections under study were suitable in terms of growth conditions such as irrigation, nutrition, and pest and disease control, and the trees were mature, healthy, and in full cropping.

### 2.2 The characteristics evaluated

The number of 50 replicates was used to evaluate the traits related to leaf, nut, and kernel. A digital caliper was used to measure leaf, nut, and kernel dimensions. Also, an electronic scale with an accuracy of 0.01 g was used to measure the weight of nuts and kernels. Almond descriptor (International Plant Genetic Resources Institute, IPGRI, Gulcan, 1985) was used to estimate qualitative traits and based on that traits were coded and ranked.

### 2.3 Statistical analysis

Analysis of variance (ANOVA) using SAS software (SAS® Procedures, 1990) was used to determine the phenotypic variation among genotypes based on the recorded traits. Pearson correlation coefficients with Student's t‐distribution test were used to determine the relationship between the recorded traits using SPSS software (SPSS Inc.; Norusis, 1998). To identify the most important influencing traits in the grouping of genotypes, principal component analysis (PCA) was applied using SPSS software. Hierarchical cluster analysis (HCA) based on Ward's method and Euclidean distance coefficients using PAST software (Hammer et al., 2001) was used to classify genotypes. The first and second principal components (PC1/PC2) were used to draw a two‐dimensional plot by determining the distribution of genotypes. Multiple regression analysis (MRA) with “stepwise linear” method was used to determine the effect of independent traits on kernel weight as a dependent trait, using SPSS software.

## 3 RESULTS AND DISCUSSION

### 3.1 Characterization of the 198 genotypes investigated

Significant variabilities were exhibited among the genotypes investigated based on the recorded traits (ANOVA, *p* < .01). The coefficient of variation (CV) ranged from 11.25 (in kernel length) to 201.79% (in blanked nuts percentage). Asgari and Khadivi (2021) reported the range from 8.81 (in nut diameter) to 272.58% (in blanked nuts percentage).

Full blooming dates (50% of flowers completely open) ranged from mid‐March to mid‐April. Tree growth vigor, tree height, trunk diameter, and canopy density were predominantly low in the majority of genotypes (Table 2). Branch leaf length ranged from 30.93 to 100.49 mm, branch leaf width varied from 12.01 to 44.94 mm, and branch petiole length ranged from 6.68 to 29.53 mm. Spur leaf length ranged from 21.06 to 65.72 mm, spur leaf width varied from 7.74 to 24.48 mm, and spur petiole length varied between 5.11 and 27.03 mm (Table 1).

**TABLE 1:** Statistical descriptive parameters for morphological traits used to study almond genotypes.

No.	Character	Abbreviation	Unit	Min	Max	Mean	SD	Coefficient of variation (%)
1	Flower date	FlDa	Date	1	7	3.56	1.49	41.94
2	Tree growth vigor	TrGrVi	Code	1	5	2.47	1.65	66.88
3	Tree height	TrHe	Code	1	5	2.67	1.72	64.38
4	Trunk color intensity	TrCoIn	Code	1	5	3.63	1.25	34.35
5	Trunk diameter	TrDi	Code	1	7	2.49	1.75	70.40
6	Canopy density	CaDe	Code	1	5	2.78	1.73	62.27
7	Branching	Br	Code	1	5	2.97	1.70	57.37
8	Branch density	BrDe	Code	1	5	3.04	1.65	54.24
9	Branch flexibility	BrFl	Code	1	5	3.10	1.27	40.97
10	Branch leaf length	BrLLe	mm	30.93	100.49	64.35	12.76	19.83
11	Branch leaf width	BrLWi	mm	12.01	44.94	20.42	4.77	23.37
12	Branch petiole length	BrPeLe	mm	6.68	29.53	18.54	4.11	22.17
13	Spur leaf length	SpLLe	mm	21.06	65.72	43.26	7.69	17.78
14	Spur leaf width	SpLWi	mm	7.74	24.48	13.21	2.58	19.53
15	Spur petiole length	SpPeLe	mm	5.11	27.03	13.02	3.33	25.61
16	Leaf density	LDe	Code	1	5	2.85	1.78	62.42
17	Leaf shape	LSh	Code	1	7	5.58	2.11	37.76
18	Leaf upper surface color	LUpSuCo	Code	1	3	2.42	0.91	37.52
19	Leaf lower surface color	LLoSuCo	Code	1	3	2.10	1.00	47.48
20	Leaf apex shape	LApSh	Code	1	3	1.02	0.20	19.61
21	Harvest date	HaDa	Date	1	3	1.81	0.98	54.36
22	Nut apex shape	NuApSh	Code	1	3	2.24	0.97	43.44
23	Nut shape	NuSh	Code	1	7	4.26	1.81	42.58
24	Nut length	NuLe	mm	22.34	45.69	34.19	4.19	12.25
25	Nut width	NuWi	mm	14.07	27.92	20.21	2.49	12.33
26	Nut thickness	NuTh	mm	8.03	21.43	14.16	1.89	13.34
27	Nut weight	NuWe	g	1.88	10.44	3.98	1.20	30.09
28	Shell hardness	SheHa	Code	1	9	4.96	1.52	30.67
29	Shell color intensity	SheCoIn	Code	1	7	4.34	1.60	36.80
30	Shell thickness	SheTh	mm	2.26	4.98	3.33	0.57	17.12
31	Kernel length	KeLe	mm	16.73	27.71	22.15	2.49	11.25
32	Kernel width	KeWi	mm	8.48	15.26	11.35	1.29	11.40
33	Kernel thickness	KeTh	mm	3.10	7.37	4.76	0.73	15.27
34	Kernel weight	KeWe	g	0.35	1.62	0.70	0.18	26.17
35	Kernel Shape	KeSh	Code	1	7	3.89	1.37	35.30
36	Kernel color intensity	KeCoIn	Code	1	9	4.62	1.36	29.37
37	Kernel shriveling	KeShr	Code	1	5	1.71	1.10	64.15
38	Kernel pubescence	KePu	Code	0	1	0.77	0.42	54.55
39	Kernel taste	KeTa	Code	1	7	4.35	1.92	44.11
40	Double kernel percentage	DoKePer	%	0	90	13.18	17.93	136.00
41	Kernel blanking percentage	KeBlPer	%	0	40	3.13	6.32	201.79

Nut and kernel shapes were predominantly oval (125 and 90 genotypes, respectively). The nut‐related characters ranged as follows: nut length: 22.34–45.69 mm, nut width: 14.07–27.92 mm, nut thickness: 8.03–21.43 mm, nut weight: 1.88–10.44 g, and shell thickness: 2.26–4.98 mm. Sakar (2019) studied five almond cultivars from northern Morocco and reported the range of 30.92–34.14 mm for nut length, 20.84–23.87 mm for nut width, and 14.22–16.57 mm for nut thickness. Also, Sakar et al. (2020) reported the range of 2.72–4.57 g for nut weight in five almond cultivars from northern Morocco.

Shell hardness was moderate in most of the genotypes (117). One of the most important goals in almond breeding programs is shell softness. The resistance to pests and diseases is higher in hard‐shell genotypes than in soft‐shell genotypes (Gradziel & Martinez‐Gomez, 2002; Khadivi‐Khub & Etemadi‐Khah, 2015; Ledbetter & Shonnard, 1992). Therefore, the type of genotype used has a considerable effect on the breeding programs, so that in the case of soft‐shell genotypes, identification of individuals that are resistant to fungi and insects is a priority, while in the case of semi‐soft‐shell genotypes, breeders seek to select genotypes having an acceptable shelling (Khadivi, Goodarzi, & Sarkhosh, 2019).

Kernel color intensity showed high diversity, including very light (4 genotypes), light (55), moderate (115), dark (23), and very dark (1). Light kernel color is preferred (Kodad et al., 2015; Socias i Company et al., 2008). A greater pubescence is associated with darker kernel color and is less desirable for nuts consumed raw (Kodad et al., 2015; Socias i Company et al., 2008).

The kernel‐related characters ranged as follows: kernel length: 16.73–27.71 mm, kernel width: 8.48–15.26 mm, kernel thickness: 3.10–7.37 mm, and kernel weight: 0.35–1.62 g. Sakar (2019) studied five almond cultivars from northern Morocco and reported the range of 22.64–24.45 mm for kernel length, 12.82–14.35 mm for kernel width, and 6.40–7.22 mm for kernel thickness. Also, Sakar et al. (2020) reported the range of 0.82–1.12 g for kernel weight in five almond cultivars from northern Morocco.

A range of 0.00%–90.00% with a mean of 13.18% was recorded for double kernel percentage. If there are two fertilized ovules in the almond and then grow and develop, double kernels occur. Double kernel is a negative phenomenon that causes kernel deformation and reduces its commercial value (Kodad et al., 2015). Also, the degree of double kernel has a significant effect on crop quality and marketing (Kester & Gradziel, 1996). Various factors, such as environmental conditions, especially low temperature in the pre‐flowering phase, are significantly influential in the occurrence of this negative phenomenon. To reduce this phenomenon, the plant nutrition program should be normal and genotypes sensitive to this phenomenon should be cultivated in milder areas in terms of climate (Kester & Gradziel, 1996).

A range of 0.00%–40.00% with a mean of 3.13% was recorded for the amount of blanked nuts. This phenomenon is greatly influenced by the fertility rate. Therefore, to reduce this phenomenon, great care should be taken in pollination, fertility, placement of bee hives, and selection of pollinizer in the orchards (Khadivi‐Khub & Etemadi‐Khah, 2015). Kernel taste was significantly variable, including bitter (38 genotypes), relatively bitter (19), sweet (110), and very sweet (31) (Table 2).

**TABLE 2:** Frequency distribution for the measured qualitative morphological characteristics in the studied almond genotypes.

Character	Frequency (no. of genotypes)					
	0	1	3	5	7	9
Flower date	—	Mid‐March (24)	Late March (106)	Early April (57)	Mid‐April (11)	—
Tree growth vigor	—	Low (100)	Moderate (50)	High (48)	—	—
Tree height	—	Small (92)	Moderate (47)	Large (59)	—	—
Trunk color intensity	—	Gray (17)	Copper (102)	Black (79)	—	—
Trunk diameter	—	Low (105)	Moderate (40)	High (51)	Very High (2)	—
Canopy density	—	Low (86)	Moderate (48)	High (64)	—	—
Branching	—	Low (70)	Moderate (55)	High (70)	—	—
Branch density	—	Low (65)	Moderate (64)	High (69)	—	—
Branch flexibility	—	Low (35)	Moderate (118)	High (45)	—	—
Leaf density	—	Low (86)	Moderate (41)	High (71)	—	—
Leaf shape	—	Wide (14)	Wide oblong (47)	Elongated narrow (5)	Oblong (132)	—
Leaf upper surface color	—	Light green (57)	Green (141)	—	—	—
Leaf lower surface color	—	Light green (89)	Green (109)	—	—	—
Leaf apex shape	—	Sharp (196)	Blate (2)	—	—	—
Harvest date	—	Early August (118)	Mid‐August (80)	—	—	—
Nut apex shape	—	Sharp (75)	Blate (123)	—	—	—
Nut shape	—	Round (2)	Oval (125)	Hearty (15)	Elongated oval (56)	—
Shell hardness	—	Very hard (4)	Hard (40)	Moderate (117)	Soft (30)	Paper (7)
Shell color intensity	—	Very light (14)	Light (64)	Moderate (93)	Dark (27)	—
Kernel Shape	—	Round (14)	Oval (90)	Hearty (86)	Elongated oval (8)	—
Kernel color intensity	—	Very light (4)	Light (55)	Moderate (115)	Dark (23)	Very dark (1)
Kernel shriveling	—	Low (135)	Moderate (56)	High (7)	—	—
Kernel pubescence	Absent (45)	Present (153)	—	—	—	—
Kernel taste	—	Bitter (38)	Relatively bitter (19)	Sweet (110)	Very sweet (31)	—

Significant correlations were detected between some traits as revealed using Pearson correlation coefficient analysis (Table 3). Branch leaf length showed significant and positive correlations with branch leaf width (*r* = .72, *p* ≤ .01), branch petiole length (*r* = .65, *p* ≤ .01), and spur leaf length (*r* = .23, *p* ≤ .01), in agreement with previous findings in almond (Asgari & Khadivi, 2021; Khadivi, Goodarzi, & Sarkhosh, 2019; Khadivi, Safdari, et al., 2019; Khadivi‐Khub & Etemadi‐Khah, 2015). Spur leaf length was positively and significantly correlated with spur leaf width (*r* = .64, *p* ≤ .01) and spur petiole length (*r* = .65, *p* ≤ .01). Nut weight showed significant and positive correlations with nut length (*r* = .70, *p* ≤ .01), nut width (*r* = .76, *p* ≤ .01), nut thickness (*r* = .73, *p* ≤ .01), and shell thickness (*r* = .65, *p* ≤ .01), in agreement with previous findings obtained between the nut‐related characters of almond (Asgari & Khadivi, 2021; Khadivi, Goodarzi, & Sarkhosh, 2019; Khadivi, Safdari, et al., 2019; Khadivi‐Khub & Etemadi‐Khah, 2015; Khadivi‐Khub & Osati, 2015; Sepahvand et al., 2015). Kernel weight was positively and significantly correlated with nut length (*r* = .69, *p* ≤ .01), nut width (*r* = .63, *p* ≤ .01), nut thickness (*r* = .63, *p* ≤ .01), nut weight (*r* = .77, *p* ≤ .01), shell thickness (*r* = .43, *p* ≤ .01), kernel length (*r* = .72, *p* ≤ .01), kernel width (*r* = .65, *p* ≤ .01), and kernel thickness (*r* = .50, *p* ≤ .01), in agreement with previous findings obtained between the kernel‐related characters of almond (Asgari & Khadivi, 2021; Khadivi, Goodarzi, & Sarkhosh, 2019; Khadivi, Safdari, et al., 2019; Khadivi‐Khub & Etemadi‐Khah, 2015; Khadivi‐Khub & Osati, 2015; Sepahvand et al., 2015).

**TABLE 3:** Simple correlations among the morphological variables utilized in the studied almond genotypes.

Character	BrLLe	BrLWi	BrPeLe	SpLLe	SpLWi	SpPeLe	NuLe	NuWi	NuTh	NuWe	SheTh	KeLe	KeWi	KeTh	KeWe
BrLLe	1														
BrLWi	.72**	1													
BrPeLe	.65**	.52**	1												
SpLLe	.23**	.05	.11	1											
SpLWi	.13	.23**	.08	.64**	1										
SpPeLe	.13	.01	.26**	.65**	.60**	1									
NuLe	.03	.01	.00	.06	.01	.1	1								
NuWi	.07	.02	.08	.15*	.15*	.16*	.59**	1							
NuTh	.01	−.03	.07	.13	.15*	.16*	.50**	.79**	1						
NuWe	.09	.02	.08	.07	.05	.12	.70**	.76**	.73**	1					
SheTh	−.08	−.15*	−.06	.09	.07	.15*	.38**	.66**	.71**	.65**	1				
KeLe	.06	.01	−.03	.06	−.04	.05	.83**	.43**	.39**	.63**	.27**	1			
KeWi	.06	−.06	.04	.15*	.11	.15*	.46**	.80**	.65**	.67**	.60**	.43**	1		
KeTh	.02	.06	.05	.02	.06	.02	.04	.13	.39**	.21**	.20**	.19**	.27**	1	
KeWe	.13	.11	.07	.08	.07	.12	.69**	.63**	.63**	.77**	.43**	.72**	.65**	.50**	1

*Note*: For the explanation of morphological character symbols, see Table 1.

*, **Correlation is significant at *p* ≤ .05 and .01 levels, respectively.

Kernel size depends on kernel weight. Large kernels increase the marketable value of almond because they meet the standards of quality required by the market (Socias i Company et al., 2008). Thus, identifying the characters that improve the kernel size is important. Thus, the effect of independent traits on kernel weight as a dependent trait was investigated with MRA (Table 4). The MRA showed that kernel weight was positively and significantly associated with nut weight, kernel thickness, kernel length, kernel width, nut length, and branch leaf width, while it was as negatively and significantly associated with shell thickness. Thus, these key variables are the main traits accounting for kernel weight, and they should be considered together in breeding with aiming increasing kernel weight. Significant regression associations between kernel weight and nut and kernel‐related characters have been previously reported with MRA in almond (Asgari & Khadivi, 2021; Khadivi, Safdari, et al., 2019; Sepahvand et al., 2015). In addition, kernel weight and others geometrical traits were investigated by Ledbetter and Sisterson (2010) to determine best regression models that could describe variations in almond kernel weight. Also, Sakar, Yamani, Boussakouran, & Rharrabti, 2019; Sakar, Yamani, & Rharrabti, 2019 revealed that nut and kernel length was the most variable that explained variations in the majority of calculated traits.

**TABLE 4:** The traits associated with kernel weight in the almond genotypes studied as revealed using multiple regression analysis and coefficients.

Dependent character	Independent character	*R*	*r* ^2^	Standardized beta coefficients	*t* Value	*p* Value
Kernel weight	Nut weight	.77 a	.60	.37	6.35	.00
	Kernel thickness	.84 b	.72	.34	10.25	.00
	Kernel length	.88 c	.79	.18	3.04	.00
	Kernel width	.89 d	.80	.21	4.76	.00
	Nut length	.90 e	.81	.22	3.48	.00
	Shell thickness	.91 f	.82	−.13	−2.80	.01
	Branch leaf width	.92 g	.83	.07	2.14	.03

The PCA showed that the first 14 components accounted for 78.09% of the total variance (Table 5). The characters, including nut width, nut thickness, nut weight, shell thickness, kernel width, and kernel weight, were correlated with PC1, accounting for 13.86% of the total variance. The traits related to nut and kernel size are important for differentiating the almond genotypes (Khadivi‐Khub & Etemadi‐Khah, 2015; Sakar et al., 2020; Sakar, Yamani, & Rharrabti, 2019). The PC2 included tree growth vigor, canopy density, branching, branch density, leaf density, accounting for 12.28% of the total variance. Three characters, including branch leaf length, branch leaf width, and branch petiole length, formed the PC3, accounting for 5.86% of the total variance. PCA has been previously used to investigate the phenotypic diversity of almond genotypes (Asgari & Khadivi, 2021; Khadivi, Goodarzi, & Sarkhosh, 2019; Khadivi, Safdari, et al., 2019; Sakar et al., 2020; Sakar, Yamani, & Rharrabti, 2019; Sepahvand et al., 2015) and other plants (Khadivi et al., 2021; Khadivi & Mirheidari, 2021; Khadivi, Mirheidari, & Moradi, 2022; Khadivi, Mirheidari, Saeidifar, & Moradi, 2022; Moradi et al., 2022; Safdari & Khadivi, 2021).

**TABLE 5:** Eigenvalues of the principal component axes from the principal component analysis of the morphological characters in the studied almond genotypes.

Character	Component													
	1	2	3	4	5	6	7	8	9	10	11	12	13	14
Flower date	−.20	.00	.27	.52	−.04	.01	.15	.09	.15	−.39	−.05	−.20	−.09	.08
Tree growth vigor	.08	.74**	.13	.00	−.01	.03	.01	.13	.02	−.12	−.13	.12	.05	.19
Tree height	−.07	.56	.01	.68**	.06	.01	−.09	.12	−.08	−.04	.02	−.03	−.01	.02
Trunk color intensity	−.06	.42	.03	.72**	−.06	−.08	−.01	.05	−.09	−.03	−.03	.12	.01	.05
Trunk diameter	−.01	.50	.04	.74**	−.08	.00	−.03	.13	−.05	.02	−.10	−.11	−.04	.01
Canopy density	.04	.91**	.04	.16	.04	.00	.07	.03	.04	−.01	.00	−.04	.04	−.01
Branching	−.08	.90**	.11	.14	.01	.06	.04	.08	.01	−.01	.05	.01	−.03	−.08
Branch density	−.05	.87**	.11	.16	−.01	.05	.03	.09	−.01	−.03	.05	.02	−.03	−.13
Branch flexibility	.20	.50	−.11	−.22	.12	−.02	−.04	−.13	−.43	.09	.07	−.07	−.21	−.21
Branch leaf length	.04	.13	.87**	.01	.11	−.05	.01	.18	.01	−.06	−.12	.10	−.06	.01
Branch leaf width	−.03	.18	.74**	.18	.04	−.04	.05	.20	−.40	−.10	−.07	.03	−.04	.14
Branch petiole length	.04	.12	.84**	.03	.10	−.04	.01	.11	.08	.07	.01	−.20	.12	−.12
Spur leaf length	.07	−.02	.09	−.03	.88**	.03	−.02	.01	.08	.06	−.08	.12	−.04	−.02
Spur leaf width	.06	.09	.02	.03	.85**	−.11	.04	.03	−.26	−.01	−.03	.07	−.03	.04
Spur petiole length	.12	−.01	.09	−.04	.84**	.03	−.01	.05	.08	.03	.00	−.21	.10	−.07
Leaf density	.07	.90**	.06	.13	.01	−.02	.05	.03	.00	−.01	.02	−.03	.03	.00
Leaf shape	−.01	.05	−.11	−.06	−.03	−.04	−.04	−.08	.84**	.08	−.05	−.06	−.20	.00
Leaf upper surface color	−.05	.17	.21	.08	.08	−.01	.03	.85**	−.03	−.12	−.03	−.05	.13	.03
Leaf lower surface color	−.05	.13	.19	.09	.03	.02	.02	.86**	−.04	.00	−.03	.00	−.15	−.05
Leaf apex shape	.08	.03	.02	−.02	.02	−.03	−.07	−.02	−.15	.02	.02	.04	.90**	−.07
Harvest date	−.36	.14	.19	.34	−.09	−.16	−.01	.25	.37	−.41	−.14	−.07	.07	.00
Nut apex shape	.29	.08	−.12	−.09	.03	−.15	.19	−.16	−.01	−.04	.01	.72**	.10	.09
Nut shape	.02	.03	−.11	.00	−.02	.80**	−.06	−.03	−.01	.01	−.04	−.08	−.05	.00
Nut length	.64	.03	.02	−.04	.01	.65**	−.06	.02	−.04	−.05	−.07	−.03	.00	.12
Nut width	.91**	.03	.04	.05	.08	.00	−.06	−.03	.01	.06	−.03	.01	−.02	.01
Nut thickness	.85**	−.03	.00	−.05	.10	−.05	.25	−.03	−.01	.13	.00	.03	.11	.04
Nut weight	.89**	.08	.05	−.06	.00	.21	.08	.01	−.01	−.06	−.05	.01	.06	−.03
Shell hardness	−.28	−.06	.08	.05	−.08	.30	−.10	.18	−.10	.28	.07	.60	−.05	−.16
Shell color intensity	−.09	−.10	−.24	−.14	−.09	−.36	.16	−.01	.01	.19	.52	−.17	.10	.14
Shell thickness	.77**	−.14	−.09	−.09	.06	−.08	.05	−.15	−.02	.20	.01	−.03	.20	.02
Kernel length	.53	.10	.02	−.08	−.03	.72**	.10	.03	−.01	−.12	−.08	.14	−.02	.02
Kernel width	.85**	.02	.01	−.06	.07	−.04	.05	−.03	−.03	.09	−.01	.06	−.13	−.14
Kernel thickness	.20	.05	.02	−.03	.01	.02	.88**	.03	−.10	.08	.06	.14	−.02	−.11
Kernel weight	.75**	.13	.06	.07	.02	.35	.36	.11	−.02	−.16	.01	.07	−.08	−.06
Kernel shape	−.43	−.06	−.07	.01	−.05	.53	.26	.03	−.03	.15	.00	.07	.17	.28
Kernel color intensity	−.09	.07	−.04	.02	−.09	−.02	.04	−.04	−.10	.02	.83**	.05	.00	.01
Kernel shriveling	.18	−.09	−.01	−.05	.09	−.05	−.01	−.09	.13	.80**	.04	.06	.00	.05
Kernel pubescence	.20	−.05	−.05	−.06	.12	−.02	−.28	−.03	.23	−.39	.44	.27	−.12	.05
Kernel taste	.09	−.23	.01	.45	.12	.06	.18	−.15	.21	.05	.26	.03	.12	−.35
Double kernel percentage	.17	.08	.02	.04	.01	−.02	.87**	.01	.06	−.08	.01	−.06	−.06	−.08
Kernel blanking percentage	−.04	−.09	−.01	.04	−.02	.09	−.16	−.04	.04	.03	.09	.00	−.06	.82**
Total	5.68	5.04	2.40	2.38	2.36	2.35	2.09	1.85	1.50	1.45	1.34	1.27	1.17	1.15
% of Variance	13.86	12.28	5.86	5.81	5.76	5.72	5.10	4.50	3.66	3.53	3.27	3.09	2.84	2.81
Cumulative %	13.86	26.15	32.00	37.81	43.57	49.29	54.39	58.89	62.55	66.08	69.36	72.44	75.28	78.09

** Eigenvalues ≥0.65 are significant at the *p* ≤ .01 level.

**FIGURE 1:**
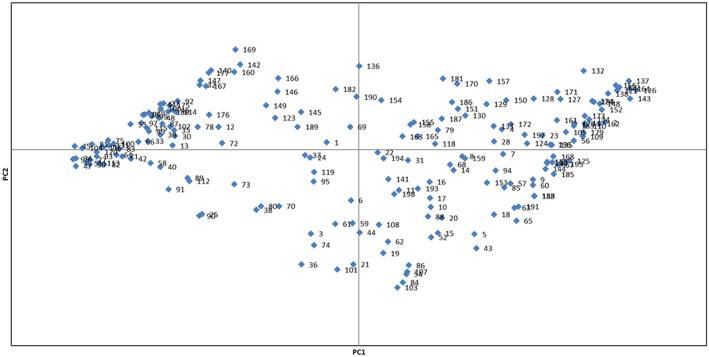
Scatter plot for the studied almond genotypes based on PC1/PC2. The numbers represent the genotypes of each area in the plot, including 1–120 (Borj) and 121–198 (Noorabad).

The scatter plot created using PC1/PC2 showed phenotypic variations among the genotypes (Figure 1). Starting from negative to positive values of PC1, the genotypes showed gradual increases in nut width, nut thickness, nut weight, shell thickness, kernel width, and kernel weight. Also, starting from negative to positive values of PC2, the characters, including tree growth vigor, canopy density, branching, branch density, showed gradual increases among the genotypes studied.

The Ward dendrogram clustered the genotypes into two major clusters (not shown). The first cluster (I) included 15 genotypes. The remaining genotypes were placed into the second cluster (II), forming four subclusters. Subcluster II‐A included 49 genotypes, and subcluster II‐A consisted of 37 genotypes. Subcluster II‐C contained 57 genotypes, while the rest 40 genotypes formed subcluster II‐D.

### 3.2 Characterization of the late‐blooming genotypes selected

After clustering the genotypes based on the full‐blooming dates, 68 late‐blooming genotypes were recognized and reanalyzed based on the quantitative characters to select the superior ones. The CV ranged from 9.75% (in kernel width) to 26.83% (in kernel weight). Branch leaf‐related characters were as follows: leaf length: 41.13–100.49 mm, leaf width: 13.81–44.94 mm, and petiole length: 12.38–28.46 mm. Spur leaf‐related characters were as follows: leaf length: 21.06–58.72 mm, leaf width: 8.60–20.50 mm, and petiole length: 7.22–20.60 mm (Table 6).

**TABLE 6:** Descriptive statistics for quantitative morphological traits utilized in the late‐blooming almond genotypes.

No.	Character	Unit	Min	Max	Mean	SD	Coefficient of variation (%)
1	Branch leaf length	mm	41.13	100.49	68.87	12.64	18.36
2	Branch leaf width	mm	13.81	44.94	22.35	4.91	21.97
3	Branch petiole length	mm	12.38	28.46	19.87	3.53	17.75
4	Spur leaf length	mm	21.06	58.72	41.99	6.51	15.51
5	Spur leaf width	mm	8.60	20.50	12.89	2.19	16.97
6	Spur petiole length	mm	7.22	20.60	12.78	2.70	21.16
7	Nut length	mm	22.34	43.05	33.04	3.95	11.95
8	Nut width	mm	14.07	24.34	19.41	2.31	11.88
9	Nut thickness	mm	9.21	18.00	13.50	1.82	13.52
10	Nut weight	g	1.88	6.62	3.57	0.94	26.29
11	Shell thickness	mm	2.26	4.59	3.05	0.52	17.01
12	Kernel length	mm	16.73	25.91	21.66	2.45	11.32
13	Kernel width	mm	8.50	13.64	10.78	1.05	9.75
14	Kernel thickness	mm	3.56	7.37	4.72	0.83	17.57
15	Kernel weight	g	0.35	1.41	0.67	0.18	26.83

**FIGURE 2:**
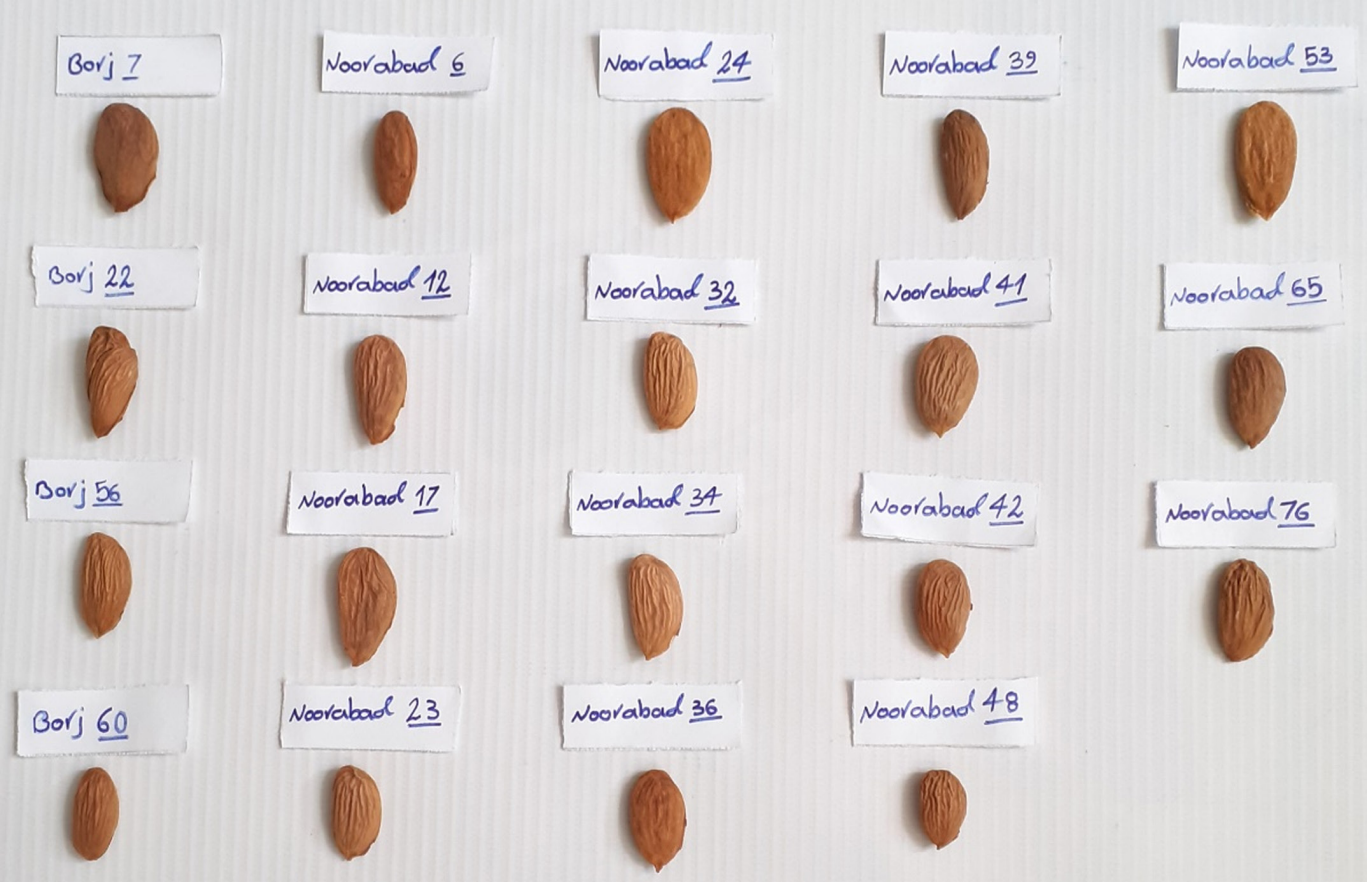
The kernels of the 19 promising late‐blooming almond genotypes identified.

Nut‐related characters were as follows: nut length: 22.34–43.05 mm, nut width: 14.07–24.34 mm, nut thickness: 9.21–18.00 mm, nut weight: 1.88–6.62 g, and shell thickness: 2.26–4.59 mm. Kernel‐related characters were as follows: kernel length: 16.73–25.91 mm, kernel width: 8.50–13.64 mm, kernel thickness: 3.56–7.37 mm, and kernel weight: 0.35–1.41 g (Table 6). Kernel weight with average of approximately 1.00 g is common in many European and American cultivars, and such weight is a desired trait in breeding programs (Kester & Gradziel, 1996). Based on ideal values of the important and commercial characters of almond such as fruit yield, nut weight, shell hardness, kernel shape, kernel weight, and kernel taste, 19 late‐blooming genotypes were promising and are recommended for cultivation in orchards. The pictures of kernels of the superior late‐blooming almond genotypes selected are shown in Figure 2.

The PCA clustered the characters into four PCs, justifying 75.77% of the total variance (Table 7). Eight characters, including nut length, nut width, nut thickness, nut weight, shell thickness, kernel length, kernel width, and kernel weight were correlated with PC1 accounting for 35.75% of the total variance. The PC2 was associated with spur leaf length, spur leaf width, and spur petiole length, accounting for 15.82% of the total variance. Three characteristics, including branch leaf length, branch leaf width, and branch petiole length, were correlated with PC3, accounting for 15.25% of the total variance.

**TABLE 7:** Eigenvalues of the principal component axes from the principal component analysis of morphological characters in the late‐blooming almond genotypes.

Character	Component			
	1	2	3	4
Branch leaf length	.00	.19	.90**	.00
Branch leaf width	−.04	.04	.85**	−.06
Branch petiole length	−.07	.08	.80**	.11
Spur leaf length	.10	.84**	.18	−.17
Spur leaf width	−.10	.84**	.10	.04
Spur petiole length	.12	.87**	.04	.06
Nut length	.82**	−.16	.05	−.42
Nut width	.82**	.14	−.09	−.13
Nut thickness	.79**	.21	−.05	.16
Nut weight	.91**	.02	−.01	.07
Shell thickness	.64**	.10	−.21	.17
Kernel length	.81**	−.22	.03	−.11
Kernel width	.82**	.13	−.02	.29
Kernel thickness	.19	−.07	.05	.92**
Kernel weight	.86**	−.07	.09	.33
Total	5.36	2.37	2.29	1.34
% of Variance	35.75	15.82	15.25	8.96
Cumulative %	35.75	51.56	66.81	75.77

** Eigenvalues ≥0.64 are significant at the *p* ≤ .01 level.

**FIGURE 3:**
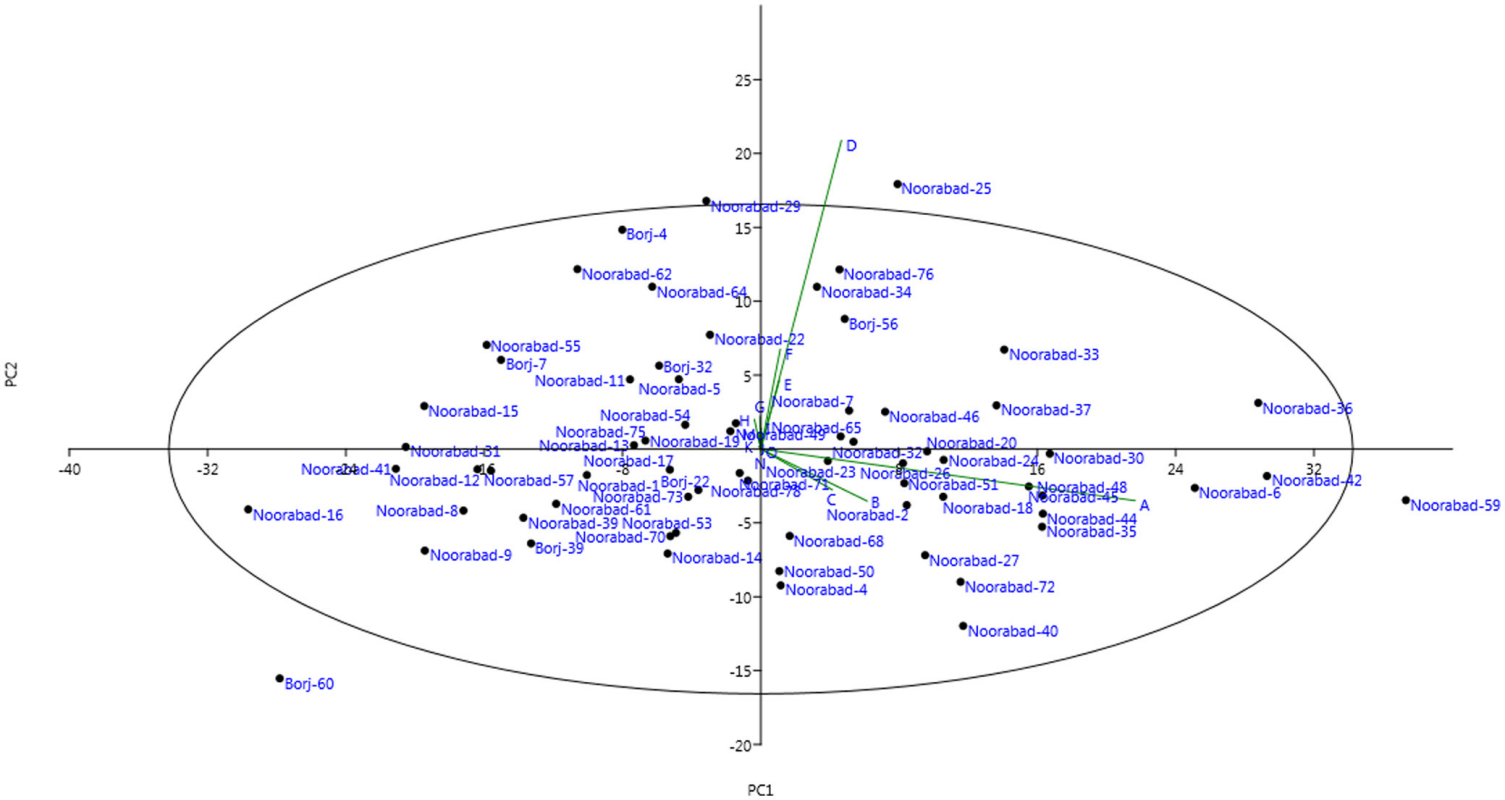
Scatter plot for the late‐blooming almond genotypes based on PC1/PC2.

**FIGURE 4:**
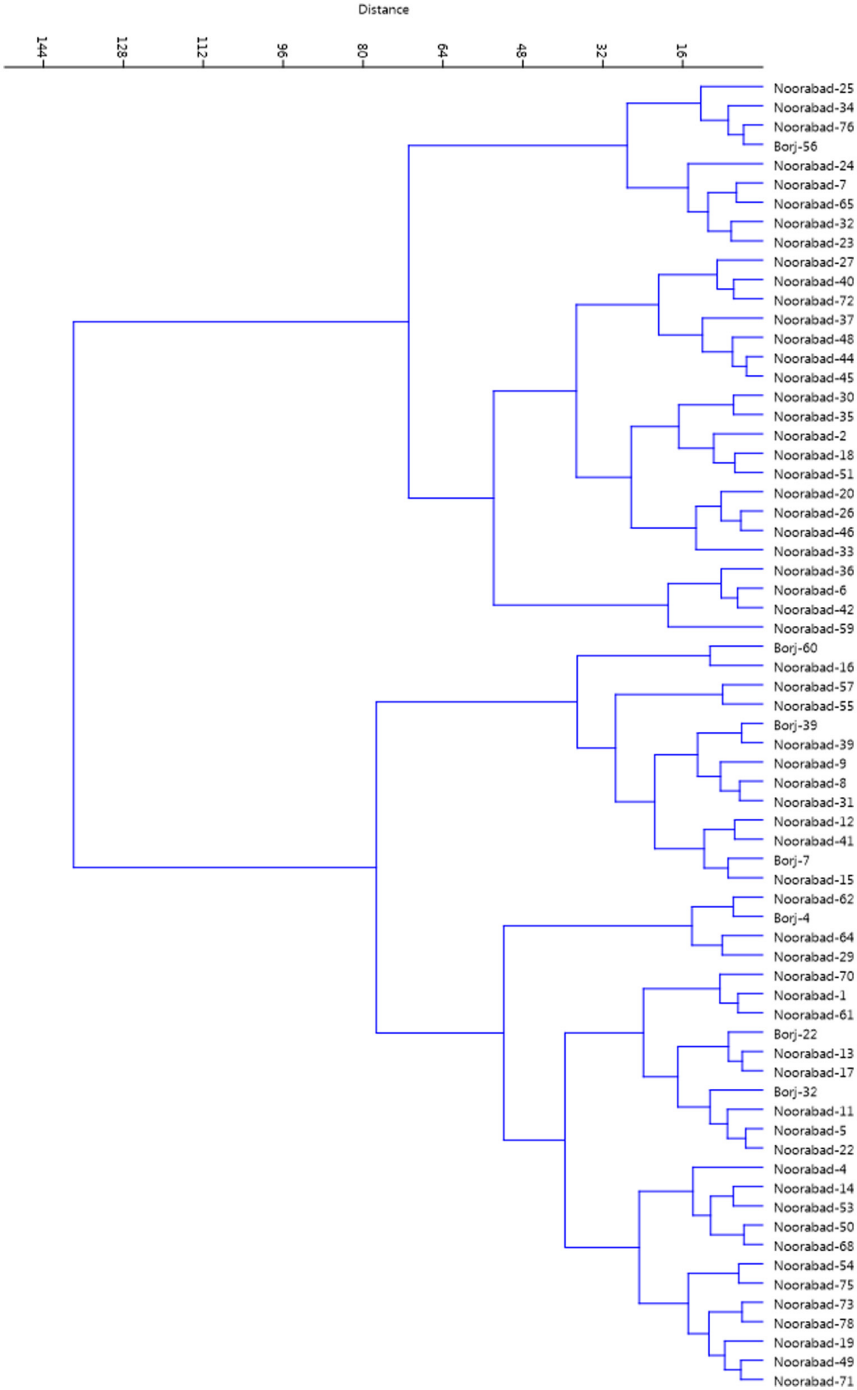
Ward cluster analysis of the late‐blooming almond genotypes based on morphological traits using Euclidean distances.

Two‐dimensional distribution of the genotypes was shown based on effective traits in the PC1 and PC2 (Figure 3). Accumulation of genotypes in one area of the plot indicated a similarity between them. The HCA based on Ward's method divided the genotypes studied into two main clusters (Figure 4). The first cluster (I) contained 28 genotypes. The rest 40 genotypes were classified into the second cluster (II), forming two subclusters.

One of the most important limiting factors for the distribution and production of almond in the world, including Iran is spring frost, which causes the loss of blossoms. In areas where there is frequent spring frost, the best way to avoid, the cultivation late‐blooming almond cultivars is preferred. Considering that in ancient times in Iran, almond propagation was through seeds, therefore this nut has a considerable genetic diversity and a rich genetic resource is provided, which provides many opportunities for breeding almond for different goals, including identifying late‐blooming genotypes (Asgari & Khadivi, 2021). In the present study, 68 late‐blooming genotypes among the studied germplasm were selected that can be used as parents in breeding programs, and 19 genotypes among these late‐blooming genotypes had high kernel quality that may be recommended for cultivation.

## 4 CONCLUSIONS

One of the most important improvement goals in most almond production areas is to increase spring frost resistance. Here, 68 late‐blooming genotypes were identified among the germplasm studied and among them, based on the ideal values of almond commercial traits, such as yield, nut weight, shell hardness, kernel weight, kernel taste, and kernel shape, 19 late‐blooming genotypes, including Noorabad‐24, Noorabad‐76, Noorabad‐32, Borj‐56, Noorabad‐23, Noorabad‐39, Borj‐7, Noorabad‐41, Noorabad‐53, Borj‐22, Noorabad‐34, Noorabad‐12, Borj‐60, Noorabad‐65, Noorabad‐48, Noorabad‐17, Noorabad‐36, Noorabad‐42, and Noorabad‐6, were promising and are recommended for cultivation in orchards. The cultivation of these genotypes directly in the orchards or using them in breeding programs to introduce new late‐flowering cultivars can help reduce the risk of spring frost. Also, due to the fact that the flowering of such genotypes is after the rainy season, the condition for the activity of pollinators such as honey bees is better, and as a result, they will be more efficient, and it will significantly help the ovule fertility rate and the reduction of blanked nuts.

## AUTHOR CONTRIBUTIONS

**Fatemeh Beigi:** Investigation (equal). **Ali Khadivi:** Investigation (equal); methodology (lead); supervision (lead); writing – original draft (lead); writing – review and editing (lead).

## CONFLICT OF INTEREST STATEMENT

The authors declare no conflict of interest.

## RESEARCH INVOLVING HUMAN PARTICIPANTS AND/OR ANIMALS

None.

## INFORMED CONSENT

None.

## Data Availability

The data that support the findings of this study are available from the corresponding author upon reasonable request.
